# Influence of TRPM4 rs8104571 genotype on intracranial pressure and outcomes in African Americans with traumatic brain injury

**DOI:** 10.1038/s41598-023-32819-7

**Published:** 2023-04-10

**Authors:** Joseph D. Krocker, Madeline E. Cotton, Jacob B. Schriner, Baron K. Osborn, Michael M. Talanker, Yao-Wei W. Wang, Charles S. Cox, Charles E. Wade

**Affiliations:** 1 Center for Translational Injury Research, Department of Surgery, McGovern Medical School at The University of Texas Health Science Center at Houston, 6431 Fannin St., MSB 5.204, Houston, TX 77030 USA; 2 Department of Pediatric Surgery, McGovern Medical School at The University of Texas Health Science Center at Houston, Houston, TX USA; 3 Program in Pediatric Regenerative Medicine, McGovern Medical School at The University of Texas Health Science Center at Houston, Houston, TX USA; 4 Red Duke Trauma Institute, Memorial Hermann-Texas Medical Center, Houston, TX USA

**Keywords:** Neuroscience, Medical research, Neurology

## Abstract

The TRPM4 gene codes for a membrane ion channel subunit related to inflammation in the central nervous system. Recent investigation has identified an association between TRPM4 single nucleotide polymorphisms (SNPs) rs8104571 and rs150391806 and increased intracranial (ICP) pressure following traumatic brain injury (TBI). We assessed the influence of these genotypes on clinical outcomes and ICP in TBI patients. We included 292 trauma patients with TBI. DNA extraction and real-time PCR were used for TRPM4 rs8104571 and rs150391806 allele discrimination. Five participants were determined to have the rs8104571 homozygous variant genotype, and 20 participants were identified as heterozygotes; 24 of these 25 participants were African American. No participants had rs150391806 variant alleles, preventing further analysis of this SNP. Genotypes containing the rs8104571 variant allele were associated with decreased Glasgow outcome scale-extended (GOSE) score (*P* = 0.0231), which was also consistent within our African-American subpopulation (*P* = 0.0324). Regression analysis identified an association between rs8104571 variant homozygotes and mortality within our overall population (*P* = 0.0230) and among African Americans (*P* = 0.0244). Participants with rs8104571 variant genotypes exhibited an overall increase in ICP (*P* = 0.0077), although a greater frequency of ICP measurements > 25 mmHg was observed in wild-type participants (*P* =  < 0.0001). We report an association between the TRPM4 rs8104571 variant allele and poor outcomes following TBI. These findings can potentially be translated into a precision medicine approach for African Americans following TBI utilizing TRPM4-specific pharmaceutical interventions. Validation through larger cohorts is warranted.

## Introduction

Traumatic brain injury (TBI) is common in the United States, with 3.6 million cases reported annually^1^. These injuries lead to over 60,000 deaths per year and an estimated economic burden of up to $221 billion^1,2^. Furthermore, TBI incidence is increasing and is expected to continue to be a major contributor to mortality and disability, both in the United States and globally^3^.

Following the initial TBI insult, a secondary injury driven by inflammation and edema commonly develops, with subsequent elevation of intracranial pressure (ICP) and reduced intracranial blood flow and tissue perfusion; ICP is considered an index of this secondary injury. TBI patients with intracranial hypertension have worse clinical outcomes^4–9^. Consequently, suppression of intracranial hypertension is a cornerstone of TBI management^10^.

Intracranial hypertension has been partly attributed to increased expression of membrane ion channel subunits following central nervous system (CNS) injury and subsequent activation of inflammatory mechanisms^11^. Specifically, the pore-forming membrane protein sulfonylurea receptor-1, also known as ATP binding cassette subfamily C member 8 (SUR1/ABCC8), and transient receptor potential cation channel subfamily M member 4 (TRPM4) have been implicated. Subunits of these proteins interact to produce the hetero-octamer membrane sodium transporter Sur1-NC_Ca-ATP_^11^. Sur1-NC_Ca-ATP_ is involved in maintaining electrochemical potential across the cell membrane in response to calcium imbalance^11^. However, inadvertent activation of Sur1-NC_Ca-ATP_ occurs with ATP depletion, leading to sodium influx, increased oncotic pressure, cellular swelling, and cell death within neurons^12^, astrocytes^13^, oligodendrocytes^14^, and microvascular endothelial cells^15^. TBI results in a depletion of ATP, and this physiological cascade could potentially exacerbate the secondary inflammation and elevated ICP exhibited with head injuries.

Despite multiple clinical trials, conflicting results have led to disagreement about the appropriate course of action for managing elevated ICP^16,17^. Consequently, clinicians have called for a personalized medicine approach to TBI management tailored to individual patients and to specific intracranial pathology^17,18^. Jha et al. have recently identified two TRPM4 single nucleotide polymorphisms (SNPs) associated with elevated ICP, rs8104571 and rs150391806^18^. rs8104571 is an intron variant that is associated with increased ICP and, perhaps paradoxically, improved clinical outcomes in TBI patients^18^; rs150391806 is a second TRPM4 SNP that has also been associated with intracranial hypertension. In our study, we investigated the influence of the TRPM4 rs8104571 and rs150391806 SNPs on ICP and clinical outcomes in a cohort of moderate to severe TBI patients. These polymorphisms show potential for a precision medicine approach using genotype-specific pharmaceutical intervention.

## Results

### Population genetics

Of the 695 patients meeting eligibility criteria, 294 experienced moderate or severe head injury and were included. Two participants had inadequate amounts of DNA for genotyping, leaving 292 participants for genetic analysis. Forty-seven of these participants did not have sufficient blood samples obtained in the emergency department, and subsequently, DNA extraction was performed on blood samples drawn upon intensive care unit admission. Only the wild-type rs150391806 allele was observed within our TBI cohort, preventing further analysis of this SNP. rs8104571 genotype frequencies observed in our study are compared to previously reported population frequencies in Table 1. Twenty-four of the 25 participants (96%) with at least one copy of the variant T rs8104571 allele were African American. A single Hispanic participant was heterozygous for the rs8104571 SNP. Our overall study population genotype results were not significantly divergent from Hardy–Weinberg equilibrium parameters (*P* = 0.1333), although genotype distribution more closely approximated equilibrium conditions within our African-American subpopulation (*P* = 0.8922). Compared to an African-American population residing in the Southwestern United States, there was no statistically significant difference in variant rs8104571 allele abundance among African Americans in our study (*P* = 0.0756), despite an increased variant allele frequency observed in our study (0.269 vs. 0.148)^19^.

**Table 1:** TRMP4 rs8104571 genotype and allele distribution comparison between our study, the 1000 Genomes Project, and an investigation by Jha et al., 2019, presented as frequency (n).

	N	TRMP4 rs8104571				
		Genotype frequency			Allele frequency	
		C/C	C/T	T/T	C	T
Overall population						
Current study	292	0.914 (267)	0.068 (20)	0.017 (5)	0.949	0.051
1000 genomes database^a^^19^	2,504	0.893 (2235)	0.097 (242)	0.011 (27)	0.941	0.059
Jha et al.^18^	385	0.979 (377)	0.021 (8)	0 (0)	0.990	0.010
African Ancestry						
Current study	54	0.556 (30)	0.352 (19)	0.093 (5)	0.731	0.269
1000 genomes database^b^^19^	61	0.721 (44)	0.262 (16)	0.016 (1)	0.852	0.148
Jha et al.^c^^18^	n/a	n/a	n/a	n/a	n/a	n/a

*C* cytosine, *T* thymine; ^a^Population source: an international cohort; ^b^Population source: African Americans in the Southwestern United States; ^c^There was an unknown proportion of African Americans within this primarily White study population.

### Overall population demographics, clinical characteristics, and outcomes

Demographics, clinical features, head injury characteristics, outcomes, and TRPM4 rs8104571 genotype data are detailed in Tables 2 and 3 for our overall study population. With the exception of race and ethnicity, there were no significant differences in demographics between rs8104571 genotypes. Similarly, there was not a significant difference in injury characteristics, including injury severity score, admission Glasgow coma scale score, vital signs, or metabolic derangement between these groups.

**Table 2:** Demographics, clinical characteristics, and TRMP4 rs8104571 genotype distribution for the overall study population denoted as median (IQR) or number of observations (%).

	TRMP4 rs8104571 genotype								
	N	Overall	N	C/C	N	C/T	N	T/T	*P*
Demographics									
Age	292	40 (28, 59)	267	40 (28, 59)	20	38 (25, 43)	5	39 (25, 53)	0.6246
Sex	292		267		20		5		0.9239
Female		81 (28%)		74 (28%)		6 (30%)		1 (20%)	
Male		211 (72%)		193 (72%)		14 (70%)		4 (80%)	
Race and ethnicity	292		267		20		5		< 0.0001*
Asian or Pacific Islander		12 (4%)		12 (4%)		0 (0%)		0 (0%)	
Black or African-American		54 (18%)		30 (11%)		19 (95%)		5 (100%)	
White, Hispanic or Latino		72 (25%)		71 (27%)		1 (5%)		0 (0%)	
White, non-Hispanic or Latino		154 (53%)		154 (58%)		0 (0%)		0 (0%)	
Admission clinical characteristics and labs									
Injury Severity score	292	30 (25, 41)	267	30 (25, 41)	20	30 (26, 41)	5	27 (18, 34)	0.4930
Glasgow Coma Scale	292	3 (3, 10)	267	3 (3, 12)	20	3 (3, 7)	5	3 (3, 9)	0.5198
Systolic blood pressure (mmHg)	291	110 (90, 135)	266	110 (90, 137)	20	119 (99, 126)	5	90 (65, 125)	0.4311
Heart rate (beats per minute)	292	107 (85, 128)	267	107 (85, 128)	20	104 (83, 136)	5	105 (65, 124)	0.7511
Blood transfusion requirement	292	266 (91%)	267	244 (91%)	20	17 (85%)	5	5 (100%)	0.4885
pH	285	7.24 (7.15, 7.31)	260	7.24 (7.16, 7.31)	20	7.21 (7.11, 7.29)	5	7.14 (7.11, 7.28)	0.3368
Base excess (mmol/L)	281	− 6 (− 9, − 2)	256	− 6 (− 8, − 2)	20	− 8 (− 10, − 2)	5	− 6 (− 9, − 4)	0.6504
ICU requirement	292	281 (96%)	267	257 (96%)	20	19 (95%)	5	5 (100%)	0.8694

*C* cytosine, *T* thymine, *ICU* intensive care unit, *IQR* interquartile range, *Significant *P* values < 0.05.

**Table 3:** Head injury characteristics, clinical outcomes, and TRMP4 rs8104571 genotype distribution for the overall study population denoted as median (IQR) or number of observations (%).

	TRMP4 rs8104571 genotype								
	N	Overall	N	C/C	N	C/T	N	T/T	*P*
Head injury characteristics									
Head abbreviated injury scale	292	4 (3, 5)	267	4 (3, 5)	20	5 (3, 5)	5	3 (3, 5)	0.0911
Mechanism of head injury	292		267		20		5		0.0538
Blunt		263 (90%)		243 (91%)		17 (85%)		3 (60%)	
Penetrating		29 (10%)		24 (9%)		3 (15%)		2 (40%)	
Head injury type	292		267		20		5		0.9142
Subdural hematoma		44 (15%)		42 (16%)		2 (10%)		0 (0%)	
Intraparenchymal contusion		1 (0%)		1 (0%)		0 (0%)		0 (0%)	
Subarachnoid hemorrhage		43 (15%)		39 (15%)		4 (20%)		0 (0%)	
Epidural hematoma		8 (3%)		8 (3%)		0 (0%)		0 (0%)	
Multifocal intracranial hemorrhage		118 (40%)		108 (40%)		7 (35%)		3 (60%)	
Other		78 (27%)		69 (26%)		7 (35%)		2 (40%)	
Craniectomy or craniotomy	291	47 (16%)	266	39 (15%)	20	7 (35%)	5	1 (20%)	0.0609
Decompressive craniectomy or craniotomy	292	10 (3%)	267	8 (3%)	20	2 (10%)	5	0 (0%)	0.2853
ICP monitor requirement	292	86 (30%)	267	77 (29%)	20	8 (40%)	5	1 (20%)	0.5367
Outcomes									
In-hospital mortality	292	78 (27%)	267	69 (26%)	20	6 (30%)	5	3 (60%)	0.2169
GOSE	225	4 (1, 8)	207	4 (1, 8)	15	3 (1, 6)	3	1 (1, 1)	0.0231*
GOSE < 4	225	105 (47%)	207	92 (44%)	15	10 (67%)	3	3 (100%)	0.0299*
GOSE < 5	225	120 (53%)	207	106 (51%)	15	11 (73%)	3	3 (100%)	0.0688
GOSE > 6	225	89 (40%)	207	86 (42%)	15	3 (20%)	0	0 (0%)	0.1096

*C* cytosine, *T* thymine, *ICP* Intracranial pressure, *GOSE* Glasgow outcome scale-extended, recorded on first follow-up visit, *ICU* intensive care unit; *Significant *P* values < 0.05.

Head injury characteristics were not significantly different between genotypes despite some areas of heterogeneity. For example, the incidence of penetrating injury trended higher in rs8104571 variant allele participants, although this difference was not significant (*P* = 0.0538). ICP-related data are presented in Tables 4 and 5. ICP measurements within 5 days of admission were available for 86 participants, with an average of 85 ICP measurements per participant recorded over an average of 4.2 days. Only one homozygous variant rs8104571 patient received an ICP monitor, limiting the analysis of ICP and pediatric intensity level of therapy (PILOT) scores between rs8104571 genotypes in our study. Subsequently, C/C homozygotes were compared to any participant carrying at least one copy of the variant allele (C/T or T/T) for this portion of the analysis. Median ICP was increased in participants with rs8104571 genotypes containing the variant allele (12) compared to wild-type participants (11; *P* = 0.0077). However, the frequency of ICP measurements greater than 25 mmHg was higher in the wild-type group (5.1%) compared to the variant group (2.1%; *P* =  < 0.0001). PILOT scores were not significantly different between rs8104571 wild-type and variant groups despite a trend toward higher overall and maximum scores in those with at least one copy of the variant allele (*P* = 0.0620 and *P* = 0.1428, respectively).

**Table 4:** Intracranial pressure characteristics stratified by TRMP4 rs8104571 genotype distribution for the overall study population and African Americans denoted as median (IQR) or number of observations (%).

	TRMP4 rs8104571 genotype						
	N	Overall	N	C/C	N	C/T or T/T	*P*
Overall intracranial pressure characteristics							
ICP monitor requirement	292	86 (30%)	267	77 (29%)	20	9 (36%)	0.4526
5-Day ICP (mmHg)^a^	7444 (86)	11 (7, 16)	6573 (77)	11 (7, 16)	871 (9)	12 (7, 17)	0.0077*
5-Day ICP > 25 (mm Hg)^a^	7444 (86)	352 (4.7%)	6,573 (77)	334 (5.1%)	871 (9)	18 (2.1%)	< 0.0001*
5-Day ICP maximum (mmHg)	86	23 (17, 42)	77	23 (17, 43)	9	23 (16, 37)	0.8049
5-Day ICP minimum (mmHg)	86	2 (1, 4)	77	2 (1, 4)	9	2 (1, 5)	0.9942
5-Day PILOT score^c^	363 (86)	5 (2, 8)	322 (77)	5 (2, 8)	41 (9)	6 (4, 10)	0.0620
5-Day PILOT score maximum	86	10 (6, 14)	77	10 (6, 14)	9	14 (9, 16)	0.1428
5-Day PILOT score minimum	86	2 (2, 4)	77	2 (2, 4)	9	2 (2, 5)	0.7460
African-American intracranial pressure characteristics							
ICP monitor requirement	54	15 (28%)	30	6 (20%)	24	9 (38%)	0.1537
5-Day ICP (mmHg)^b^	1311 (15)	11 (8, 16)	440 (6)	11 (8, 14)	871 (9)	12 (7, 17)	0.0341*
5-Day ICP > 25 (mmHg)^b^	1311 (15)	38 (2.9%)	440 (6)	20 (4.6%)	871 (9)	18 (2.1%)	0.0115*
5-Day ICP maximum (mmHg)	15	22 (15, 38)	6	19 (11, 62)	9	23 (16, 37)	0.8137
5-Day ICP minimum (mmHg)	15	2 (1, 4)	6	2 (1, 5)	9	2 (1, 5)	0.6686
5-Day PILOT score^d^	68 (15)	6 (3, 9)	27 (6)	6 (2, 7)	41 (9)	6 (4, 10)	0.1687
5-Day PILOT score maximum	15	13 (9, 16)	6	9 (6, 14)	9	14 (9, 16)	0.1860
5-Day PILOT score minimum	15	2 (2, 5)	6	2 (1, 5)	9	2 (2, 5)	0.4547

*C* cytosine, *T* thymine, *ICP* Intracranial pressure, recorded within 5 days of admission; ^a^N: ICP measurements (participants), Overall study population: 7444 total ICP measurements (C/C: 6573; C/T: 803; T/T: 68); ^b^N: ICP measurements (participants), African-American study population: 1,311 total ICP measurements (C/C: 440; C/T: 803; T/T: 68); PILOT: Pediatric intensity level of therapy scale, within 5 days of admission; ^c^N: PILOT scores (participants), Overall study population: 363 total PILOT scores (C/C: 322, C/T: 37, T/T: 4); ^d^N: PILOT scores (participants), African-American study population: 68 total PILOT scores (C/C: 27, C/T: 37, T/T: 4); *Significant *P* values < 0.05.

**Table 5:** Spearman correlation analysis between intracranial pressure and Glasgow outcome scale-extended stratified by TRMP4 rs8104571 genotype.

ICP vs. GOSE		
Population	Spearman ρ	*P*
Overall^a^	− 0.0550	< 0.0001*
TRMP4 rs8104571 C/C	− 0.0397	0.0024*
TRMP4 rs8104571 C/T or T/T	− 0.0618	0.0818
African Americans^b^	0.0521	0.0672
TRMP4 rs8104571 C/C	0.4203	< 0.0001*
TRMP4 rs8104571 C/T or T/T	− 0.0618	0.0818

*C* cytosine, *T* thymine, *ICP* Intracranial pressure, recorded within 5 days of admission; ^a^Overall study population: 7444 total ICP measurements (C/C: 6573; C/T: 803; T/T: 68); ^b^African-American study population: 1311 total ICP measurements (C/C: 440; C/T: 803; T/T: 68); *GOSE* Glasgow outcome scale-extended, recorded on first follow-up visit; *Significant *P* values < 0.05.

Seventy-eight participants died during the initial hospital admission, and follow-up data were available for 147 survivors, leaving 225 participants for outcome analysis. GOSE scores were highest in rs8104571 wild-type homozygous participants, lower in heterozygotes, and lowest in the homozygous variant group with median scores of 4, 3, and 1, respectively (*P* = 0.0231). The most severe outcomes with GOSE score < 4 were more frequent in the homozygous variant participants (3/3; 100%) than in heterozygotes (10/15; 67%) and homozygous wild-type participants (92/207; 44%; *P* = 0.0299). In-hospital mortality was not significantly different between genotypes in our overall cohort, despite observations of at least double the frequency of death in participants with homozygous variant rs8104571 genotypes (3/5; 60%) compared to the wild-type group (69/267; 26%) and the heterozygote group (6/20; 30%; *P* = 0.2169). ICP was negatively correlated with GOSE score in our overall cohort (*P* =  < 0.0001), and within the wild-type group (*P* = 0.0024), but not among those carrying at least one variant allele, despite a strong trend (*P* = 0.0818; see Table 5).

Regression analysis of outcomes in participants based on TRMP4 rs8104571 genotype is illustrated in Table 6. Multiple regression analysis did not reveal an association between rs8104571 variant genotype and diminished GOSE score (*P* = 0.0708) or mortality (*P* = 0.0701), despite a strong trend. The homozygous variant rs8104571 genotype was more closely associated with poor outcomes, as evidenced by significant differences in in-hospital mortality (*P* = 0.0230) and GOSE (*P* = 0.0281) when comparing the T/T genotype to all other genotypes. Favorable and severe outcomes based on GOSE scores < 4, < 5, and > 6 could not be assessed by multiple logistic regression due to complete separation of the model secondary to an absence of favorable outcomes among those with T/T genotype.

**Table 6:** TRMP4 rs8104571 genotype and outcome analysis utilizing multiple logistic and linear regression controlling for age, sex, injury severity score, and head abbreviated injury scale.

TRMP4 rs8104571 genotype and β-coefficients					
		C/C	C/T	T/T	*P*
Overall population	In-hospital mortality	− 0.7869 (− 1.6384, 0.0528)	− 1.0034 (− 2.0760, 0.0169)	–	0.0701
	GOSE score	1.2551 (0.1722, 2.3379)	0.7779 (− 0.5164, 2.0721)	–	0.0708
African Americans	In-hospital mortality	− 0.8928 (− 2.1327, 0.1863)	− 1.0099 (− 2.3305, 0.1409)	–	0.0785
	GOSE score	1.5325 (0.0744, 2.9906)	0.5602 (− 0.9915, 2.1118)	–	0.1071

		C/C	C/T or T/T	*P*	
Overall population	In-hospital mortality	− 1.1449 (− 0.6409, 0.3676)	–	0.5722	
	GOSE score	0.4756 (− 0.1497, 1.0988)	–	0.1355	
African Americans	In-hospital mortality	− 0.2184 (− 0.9203, 0.4631)	–	0.5288	
	GOSE score	0.7323 (− 0.2186, 1.6832)	–	0.1272	

		C/C or C/T	T/T	*P*	
Overall population	In-hospital mortality	− 1.2936 (− 2.4423, − 0.1857)	–	0.0230*	
	GOSE score	1.6233 (0.1759, 3.0707)	–	0.0281*	
African Americans	In-hospital mortality	− 1.4202 (− 2.9300, − 0.1773)	–	0.0244*	
	GOSE score	1.6215 (− 0.0697, 3.3127)	–	0.0597	

*C* cytosine, *T* thymine, *GOSE* Glasgow outcome scale-extended, recorded on first follow-up visit. GOSE scores < 4, < 5, and > 6 could not be assessed by multiple logistic regression due to an absence of favorable outcomes among those with the T/T genotype resulting in unstable parameter estimates; *Significant *P* values < 0.05.

### African-American subpopulation demographics, clinical characteristics, and outcomes

Demographics, clinical features, head injury characteristics, outcomes, and TRPM4 rs8104571 genotype data are described in Tables 7 and 8 for our African-American subpopulation. This TBI cohort contained 54 African-American participants and included 24 of the 25 participants with at least one rs8104571 variant allele. Overall demographics and injury characteristics were not significantly different between rs8104571 genotypes.

**Table 7:** Demographics, clinical characteristics, and TRMP4 rs8104571 genotype distribution for African Americans within the study population denoted as median (IQR) or number of observations (%).

	TRMP4 rs8104571 genotype								
	N	Overall	N	C/C	N	C/T	N	T/T	*P*
Demographics									
Age	54	38 (27, 54)	30	37 (27, 59)	19	38 (24, 43)	5	39 (25, 53)	0.9818
Sex	54		30		19		5		0.7908
Female		13 (24%)		6 (20%)		6 (32%)		1 (20%)	
Male		41 (76%)		24 (80%)		13 (68%)		4 (80%)	
Admission clinical characteristics and labs									
Injury severity score	54	30 (26, 38)	30	34 (26, 38)	19	29 (26, 38)	5	27 (18, 34)	0.4340
Glasgow coma scale	54	3 (3, 7)	30	3 (3, 9)	19	3 (3, 4)	5	3 (3, 9)	0.8788
Systolic blood pressure (mm Hg)	54	110 (90, 127)	30	105 (90, 134)	19	118 (98, 122)	5	90 (65, 125)	0.5107
Heart rate (beats per minute)	54	103 (83, 130)	30	102 (86, 131)	19	102 (82, 125)	5	105 (65, 124)	0.8632
Blood transfusion requirement	54	47 (87%)	30	26 (87%)	19	16 (84%)	5	5 (100%)	0.6431
pH	54	7.23 (7.14, 7.30)	30	7.23 (7.19, 7.32)	19	7.21 (7.10, 7.29)	5	7.14 (7.11, 7.28)	0.4704
Base excess (mmol/L)	54	− 7 (− 9, − 2)	30	− 6 (− 9, − 2)	19	− 7 (− 10, − 1)	5	− 6 (− 9, − 4)	0.9344
ICU requirement	54	51 (94%)	30	28 (93%)	19	18 (95%)	5	5 (100%)	0.8320

*C* cytosine,* T* thymine,* ICU* intensive care unit,* IQR* interquartile range, *Significant *P* values < 0.05.

**Table 8:** Head injury characteristics, outcomes, and TRMP4 rs8104571 genotype distribution for African Americans within the study population denoted as median (IQR) or number of observations (%).

	TRMP4 rs8104571 genotype								
	N	Overall	N	C/C	N	C/T	N	T/T	*P*
Head injury characteristics									
Head abbreviated injury scale	54	5 (3, 5)	30	4 (3, 5)	19	5 (3, 5)	5	3 (3, 5)	0.2826
Mechanism of head injury	54		30		19		5		0.5356
Blunt		43 (80%)		24 (80%)		16 (84%)		3 (60%)	
Penetrating		11 (20%)		6 (20%)		3 (16%)		2 (40%)	
Head injury type	54		30		19		5		0.9425
Subdural hematoma		5 (9%)		3 (10%)		2 (11%)		0 (0%)	
Intraparenchymal contusion		0 (0%)		0 (0%)		0 (0%)		0 (0%)	
Subarachnoid hemorrhage		6 (11%)		3 (10%)		3 (16%)		0 (0%)	
Epidural hematoma		2 (4%)		2 (7%)		0 (0%)		0 (0%)	
Multifocal intracranial hemorrhage		24 (44%)		14 (47%)		7 (37%)		3 (60%)	
Other		17 (32%)		8 (27%)		7 (37%)		2 (40%)	
Craniectomy or craniotomy	54	11 (20%)	30	3 (10%)	19	7 (37%)	5	1 (20%)	0.0546
Decompressive craniectomy or craniotomy	54	2 (4%)	30	0 (0%)	19	2 (11%)	5	0 (0%)	0.2977
ICP monitor requirement	54	15 (28%)	30	6 (20%)	19	8 (42%)	5	1 (20%)	0.2074
Outcomes									
In-hospital mortality	54	16 (30%)	30	8 (27%)	19	5 (26%)	5	3 (60%)	0.3387
GOSE	43	3 (1, 8)	26	6 (1, 8)	14	3 (1, 6)	3	1 (1, 1)	0.0324*
GOSE < 4	43	23 (53%)	26	11 (42%)	14	9 (64%)	3	3 (100%)	0.1099
GOSE < 5	43	25 (58%)	26	12 (46%)	14	10 (71%)	3	3 (100%)	0.0159*
GOSE > 6	43	16 (37%)	26	13 (50%)	14	3 (21%)	0	0 (0%)	0.1089

*C* cytosine, *T* thymine, *ICP* Intracranial pressure, *GOSE* Glasgow outcome scale-extended, recorded on first follow-up visit, *IQR* interquartile range: *Significant *P* values < 0.05.

Head injury characteristics were generally consistent with our overall study cohort, including a statistically insignificant but increased incidence of penetrating head injury in the rs8104571 homozygous variant group (*P* = 0.5356). ICP-related findings described in Table 4 did not differ significantly from our overall cohort. Correlation between ICP and GOSE among African Americans depicted in Table 5 was inconsistent with our overall cohort and incongruent between genotypes within our African-American cohort. Specifically, there was a positive correlation identified between ICP and GOSE in the rs8104571 wild-type group (*P* =  < 0.0001).

Sixteen African-American participants died, and 27 participants had follow-up data available, allowing for GOSE assessment for 43 participants. rs8104571 homozygous common allele participants had higher median GOSE (6) than heterozygotes (3) and homozygous variant participants (1; *P* = 0.0324). Severe outcomes denoted by GOSE < 5 were associated with the variant rs8104571 allele (*P* = 0.0324). Strong, albeit non-significant, trends toward higher mortality (*P* = 0.3387), poor outcome based on GOSE < 4 (*P* = 0.1099), and lack of favorable outcome based on GOSE > 6 (*P* = 0.1089) were observed in African-American participants with the T rs8104571 allele as well.

Regression analysis examining outcomes based on TRMP4 rs8104571 genotype within our African-American population is depicted in Table 6. In-hospital mortality (*P* = 0.0785) and follow-up GOSE (*P* = 0.1071) were not significantly different between genotypes, despite strong trends toward poorer outcomes in those with variant rs8104571 alleles. However, regression analysis demonstrated higher mortality (*P* = 0.0244) in those participants homozygous for the variant T allele than others. GOSE < 4, < 5, and > 6 could not be assessed by multiple logistic regression due to a lack of favorable outcomes within the T/T genotype group leading to unstable parameter estimates.

Comparison of ICP-related metrics and clinical outcomes between African Americans and non-African Americans is presented in Supplementary Table 1. There were no significant differences between African Americans and non-African Americans, with the exception of ICP measurements > 25 mmHg, which were less common in African Americans (2.9%) than those of other races and ethnicities (5.1%; *P* = 0.0006).

## Discussion

The identification of two SNPs associated with elevated ICP following TBI and supported by a putative underlying biomolecular etiology related to inflammation and cell death served as the impetus for our investigation. In our study, we found an association between the TRPM4 rs8104571 variant allele and poor outcomes based on GOSE score and mortality. These associations persist within our isolated African-American cohort. Participants with rs8104571 variant genotypes were associated with an overall increase in ICP, while a higher frequency of ICP greater than 25 mm Hg was observed in wild-type participants. While our small pilot study requires validation in a larger cohort, these associations suggest that the rs8104571 variant genotype, primarily found in African Americans, may influence outcomes of TBI.

### Frequency of rs8104571 in Houston trauma population

Variant rs8104571 allele frequencies as high as 0.247 have been previously reported in populations with African ancestry^19^. We report an rs8104571 variant allele frequency of 0.269, which is 12 percentage points greater than the frequency observed in a sample population of African Americans residing in the Southwestern United States^19^. Despite a lack of statistical significance, this difference represents a strong trend toward increased variant allele abundance in the greater Houston area. This potential increase in the variant allele abundance in the Houston African-American trauma population may represent migration patterns and founder effects specific to the local area. There were no variant TRPM4 rs150391806 alleles detected in our study population. This is not necessarily unexpected as the variant rs150391806 allele is rare in United States populations: an absence of variant allele carriers has been reported in U.S. Whites, African Americans, and Gujarati Indians, and an allele frequency of just 0.008 has been reported among those with Mexican ancestry^19^.

### rs8104571 genotype and TBI outcomes

In our overall study population, we found that the variant rs8104571 allele was associated with poorer overall outcomes based on GOSE. Unfortunately, due to a lack of favorable outcomes in the T/T genotype group, regression analysis could only corroborate this finding when comparing rs8104571 variant homozygous participants against all other genotypes. However, regression analysis did identify an increase in mortality in African Americans with T/T genotype, and a strong overall trend toward poorer outcomes for those with the variant rs8104571 allele was apparent across all regression models despite a lack of significance in some cases. The identification of poor outcomes associated with the T/T genotype would typically imply recessive inheritance of the clinical phenotype associated with variant rs8104571. However, a closer look at overall trends suggests that the negative influence on outcome associated with rs8104571 may be due to codominant inheritance. Heterozygotes appear to trend toward worse outcomes than wild-type participants, and homozygous variant participants appear to have worse outcomes than heterozygotes. However, definitive conclusions regarding the negative effect on outcome observed in rs8104571 T/T homozygotes require further investigation in a larger cohort.

Our findings do not agree with the associations between rs8104571 heterozygotes and improved GOSE reported by Jha et al.^18^. These authors hypothesized that the pro-inflammatory phenotype associated with the rs8104571 variant might provide a neuroprotective benefit^18^, while our results reported herein suggest a survival disadvantage. The discrepancy is likely related to nonuniform regression analysis methods used by Jha et al.^18^. Our study did not incorporate variables including herniation, midline shift, and proportion of ICP greater than 25 mmHg because increased CNS inflammation related to rs8104571 can potentially contribute to these findings. Sample size may also be a contributing factor, as the study population in Jha et al. was primarily White, with only eight rs8104571 heterozygous participants and an absence of rs8104571 homozygous variant participants^18^.

### rs8104571 genotype and ICP

Previous investigation by Jha et al. identified an association between elevated ICP and rs8104571 heterozygous genotype compared to wild-type^18^. We do not reproduce this finding in our study, where, despite a modest increase in overall ICP within the rs8104571 variant group, the highest proportion of ICP measurements greater than 25 mm Hg was observed in wild-type participants. Further, we report improved outcomes in the wild-type group despite an overall increase in clinically significant ICP elevation compared to those with the rs8104571 variant allele. Finally, we find correlation between ICP and GOSE to be inconsistent across subgroups.

These findings may reflect the limitations of ICP as a surrogate for CNS inflammation and secondary injury within our TBI cohort. TBI clinical practice preferences at our institution favor an early intervention approach to ICP management, including a low threshold for decompressive craniectomy based on clinical suspicion and prevention of intracranial hypertension. Those at the highest risk for elevated ICP are likely to receive upfront decompressive craniectomy, and, thus, the highest ICPs are likely excluded from the analysis.

Alternatively, the resulting rs8104571 variant phenotype may result in decreased ICP and poor clinical outcome following TBI related to inflammatory pathways that do not necessarily involve intracranial hypertension. A larger sample size is required to better characterize the influence of rs8104571 genotype on ICP, although even a larger sample size may not be able to account for confounding secondary to current TBI management protocols focused on the prevention of intracranial hypertension.

### rs8104571 genotype and TBI outcome within an African-American cohort

Indicators of poor clinical outcomes associated with the rs8104571 genotype in our overall study population were generally consistent across our African-American cohort. Previous studies have postulated that social, cultural, and economic factors, as well as discrimination resulting in decreased quality of care, cause outcome disparities among African Americans compared to Whites^20^. The finding of poor outcomes associated with the rs8104571 genotype within the African-American subpopulation is an important distinction, as it suggests that genetic influence may be a contributing factor.

Clinical outcome is the result of genetic predisposition, environmental factors, injury characteristics, and clinical management. The existence of social and environmental determinants of health does not preclude the presence of clinically relevant genetic factors. African-American ancestry involves selection pressures unique to local regions within Africa resulting in distinct and clinically relevant phenotypes^21,22^. Pathophysiology specific to African Americans can be successfully leveraged toward a personalized medicine approach and more effective interventions^23^. Attributing the entirety of outcome disparity to social and environmental factors without accounting for the unique biomolecular signatures specific to those with African ancestry may lead to unintentional neglect of an already vulnerable subpopulation within the United States.

The translational implications of these findings are compelling. TRPM4 rs8104571 genotyping could be completed on the day of TBI, allowing for the targeted therapy of variant allele carriers and early mitigation of the pro-inflammatory phenotype in African Americans. Candidates for pharmaceutical intervention that may mitigate the deleterious effects of the rs8104571 variant allele include the common diabetic medication glyburide, a sulfonylurea^24^. Further, we found that 44% of African-American trauma participants with moderate to severe TBI presenting to our trauma center in Houston, Texas carried at least one copy of this variant allele. This high frequency suggests that the proposed avenue of TRPM4 rs8104571-based personalized medicine has the potential to improve outcomes in a significant number of African Americans with head injuries.

### Limitations

Our exploratory study is limited by sample size, which was inadequate to evaluate the rs150391806 SNP and insufficient to fully assess associations between rs8104571 T/T genotype and ICP. Further, associations between rs8104571 genotype and outcome measures are susceptible to type II error.

The majority of our study population required blood product transfusion, and donor DNA contamination is a potential confounder in our genotyping analysis. Nucleated cells are the primary source of DNA in buffy coat samples, specifically leukocytes. Leukoreduced blood products were provided by Gulf Coast Regional Blood Center in our study, where 95% of products must meet a threshold of less than 5 × 10^6^ leukocytes per unit of blood component. A single milliliter of whole blood contains 7 × 10^6^ leukocytes, and even in cases of massive transfusion, donor DNA contamination is likely relatively insignificant. In addition, most blood draws occurred upon emergency department admission early in the hospital course before the majority of the blood transfusion volume in many cases. Nearly all variant rs8104571 alleles were identified within African-American participants, as expected, supporting the assertion that genotyping reflects the DNA of study participants rather than blood donors.

GOSE scores are ideally obtained through patient interviews rather than retrospective chart review, as was done in our study, which may limit the accuracy of our primary outcome measure. In addition, the proportion of participants with follow-up data was limited. Finally, herein, we have examined one polymorphism successfully, although the genetic influence on TBI outcome is likely polygenic. Further insight into the biomolecular drivers of TBI could be gained from additional genetic information. For example, the variant TRPM4 rs8104571 allele may have a larger influence on CNS injury when combined with the variant ABCC8 rs2237982 allele^24^.

## Conclusions

In our retrospective TBI cohort study, the TRPM4 rs8104571 variant allele was primarily identified in African Americans and found to be associated with poor outcomes. We present clinical data on TBI patients homozygous for the rs8104571 variant and identify outcome patterns potentially representing codominant inheritance, although definitive conclusions must be reserved due to sample size. Associations between rs8104571 genotype and outcome are consistent across the African-American population, suggesting that the observed clinical TBI phenotypes are ascribed to genetic influence rather than social or environmental determinants of health. These results offer a translational opportunity in a precision medicine approach for African Americans following TBI utilizing TRPM4-specific pharmaceutical interventions, including glyburide, and warrant further validation through larger cohorts.

## Methods

### Institutional review board statement

Biological sample and clinical data collection were performed as part of an ongoing study at The University of Texas Health Science Center at Houston (UTHealth Houston) with the goal of creating a deidentified biobank and registry for exploratory observations and investigations. The original protocol was approved by the Committee for the Protection of Human Subjects (Institutional Review Board) at UTHealth Houston (HSC-GEN-12-0059) and the Memorial Hermann Health System. This study was conducted in compliance with the Declaration of Helsinki and the Health Insurance Portability and Accountability Act.

Written informed consent from patients or legally authorized representatives was obtained within 72 h of admission. A waiver of consent was provided for patients who died, patients discharged within 24 h of admission, and patients without decision-making capacity and without a surrogate decision-maker following at least three attempts to obtain consent over 72 h.

### Patient population and clinical data collection

Patients ≥ 18 years presenting to the emergency department at Memorial Hermann-Texas Medical Center, a level-1 trauma center in Houston, Texas, and requiring the highest level of trauma activation between May 1, 2017, and March 31, 2022, were eligible. Patients with moderate or severe head injury defined as a head abbreviated injury scale ≥ 3 were selected retrospectively from a pool of 695 trauma patients derived from three overlapping cohorts consisting of patients requiring transfusion of at least one red-blood-cell-containing product; patients at high risk for venous thromboembolism (VTE) development; or patients that developed VTE.

Participant demographics, clinical information, and outcome data were obtained by direct observation, through our institutional trauma registry, or from medical records. ICP and PILOT scores were obtained or calculated within the first five days of admission in cases where ICP data was available. The PILOT score represents an index of ICP-directed therapeutic effort that has been shown to correlate with TBI severity and 6-month functional outcome in patients with moderate and severe TBI^25,26^. Glasgow outcome scale-extended (GOSE) scores^27^ were calculated using documentation from the initial follow-up visit where available. Research personnel performing clinical data extraction were blinded to genotype data.

### Sample collection and processing

Admission blood samples were collected for each participant upon emergency department arrival in ethylenediaminetetraacetic acid (EDTA) tubes and inverted several times. In cases where an emergency department sample could not be obtained, the sample was collected upon intensive care unit admission. Samples were centrifuged at 1800×*g* at 5 °C for 10 min twice. The buffy coat layer was isolated and stored at – 80 °C.

### DNA extraction

DNA extraction was performed with the Gentra Puregene Tissue Kit (Qiagen) with supplementary protocol *PG04: Purification of archive-quality DNA from clotted whole blood using the Gentra Puregene Tissue Kit or Gentra Puregene Mouse Tail Kit*^28^. Briefly, buffy coat samples were thawed quickly at 37 °C. Samples were added to a solution containing 5 mL of cell lysis buffer and 30 µL of proteinase K solution in a 50 mL conical tube, gently mixed, and incubated at 55 °C overnight. Samples were cooled to room temperature utilizing a 1-min ice incubation followed by the addition of 30 µL of RNase A solution and incubation at 37 °C for 15 min. Samples were cooled to room temperature utilizing a 1-min ice incubation, and 2 mL of protein precipitation solution was added. The samples were vortexed for 20 s at high speed, incubated on ice for 10 min, and centrifuged at 2000×*g* for 10 min. The supernatant was added to a 50 mL conical tube containing 6 mL of 100% isopropanol and 10 µL glycogen solution. The solution was gently mixed and centrifuged at 2000×*g* for 3 min, the supernatant was discarded, and the pellet was reconstituted in 6 mL of 70% ethanol. The solution was centrifuged at 2000×*g* for 1 min, the supernatant was discarded, and the DNA pellet was allowed to air dry for 15 min. The DNA pellet was reconstituted in 230 µL of DNA hydration solution, incubated at 65 °C for one hour, and incubated at room temperature with gentle shaking overnight. DNA samples were stored at – 80 °C.

### Real-time PCR genotyping

Real-time PCR was performed for TRPM4 rs8104571 and TRPM4 rs150391806 genotype discrimination utilizing a StepOnePlus Real-Time PCR System (Applied Biosystems) and a TaqMan SNP Genotyping Assay (Thermo Fisher Scientific) according to the manufacturer's instructions. Genotype identification was determined with TaqMan Genotyper software (Thermo Fisher Scientific). The real-time PCR assay was run in duplicate. Common and variant allele controls were confirmed by Sanger sequencing.

### Statistical analysis

The primary outcome was GOSE. This metric captures both mortality and functional status of TBI survivors. A variety of GOSE thresholds have been utilized within the TBI literature as an indicator of favorable and unfavorable outcomes^29^. GOSE scores < 4 were considered the most severe outcomes, as patients within this category that survive are incapable of any degree of independent living. GOSE < 5 was considered severe and included patients with caregiver dependence with some independent capabilities. The GOSE cutoff of < 5 is most consistent with the Glasgow outcome scale (GOS) of < 4 used in a previous TRPM4 rs8104571 analysis by Jha et al.^18^. GOSE scores > 6 were considered favorable outcomes as these patients achieved full independence. The secondary outcome was ICP. We hypothesized that participants with rs8104571 genotypes containing the variant T allele would demonstrate poorer outcomes and higher ICPs compared to wild-type participants.

Comparison between genotypes was performed with a Kruskal–Wallis test for continuous variables or a Pearson chi-squared test for categorical variables. In cases where a categorical variable had less than five observations, a Fisher exact test was performed. Correlation was calculated with a Spearman test. Genotype analysis with multiple logistic and linear regression was calculated controlling for age, sex, injury severity score, and head abbreviated injury scale and reported as β coefficient and effect likelihood ratio *P* value. Previous analysis of the rs8104571 genotype by Jha et al. was limited due to a lack of homozygous variant genotypes within the study population^18^. For this reason, regressions were run utilizing genotype, variant allele vs. other, and homozygous variant vs. other to better assess inheritance patterns. The variant rs8104571 allele is primarily found in those of African descent, and, thus, environmental and socioeconomic factors may represent a confounder in clinical outcome assessment. For this reason, analysis evaluating outcomes was performed within the isolated African-American population. All statistics were completed using SAS software JMP v16.2.0 and SAS Studio v5.2 (SAS Institute, Inc.).

## Supplementary Information

Supplementary Information.

## Data Availability

The datasets generated during and/or analyzed during our study are available from the corresponding author on reasonable request.
