# Supplementary material for: Influence of TRPM4 rs8104571 genotype on intracranial pressure and outcomes in African Americans with traumatic brain injury
Source: Sci Rep. 2023 Apr 10;13:5815. doi: 10.1038/s41598-023-32819-7 (PMC10086037; doi:10.1038/s41598-023-32819-7)
Supplement: Supplementary file 1 — Supplementary Information. [file 41598_2023_32819_MOESM1_ESM.docx]

**Supplementary Table 1.** African American vs non-African American ICP and clinical outcome characteristics denoted as median (IQR) or number of observations (%).

| **African American vs. Non-African American ICP and Clinical Outcome Characteristics** | | | | | | | |
| --- | --- | --- | --- | --- | --- | --- | --- |
|  | **N** | **Overall** | **N** | **African American** | **N** | **Non-African American** | ***P*** |
| ICP Monitor Requirement | 292 | 86 (30%) | 54 | 15 (28%) | 238 | 71 (30%) | 0.7650 |
| 5-Day ICP (mm Hg)^≠^ | 7,444 (86) | 11 (7, 16) | 1,311 (15) | 11 (8, 16) | 6,133 (71) | 11 (7, 16) | 0.0605 |
| 5-Day ICP >25 (mm Hg)^≠^ | 7,444 (86) | 352 (4.7%) | 1,311 (15) | 38 (2.9%) | 6,133 (71) | 314 (5.1%) | 0.0006* |
| 5-Day ICP Maximum (mm Hg) | 86 | 23 (17, 42) | 15 | 22 (15,38) | 71 | 26 (17, 42) | 0.6044 |
| 5-Day ICP Minimum (mm Hg) | 86 | 2 (1, 4) | 15 | 2 (1,4) | 71 | 2 (1,4) | 0.7519 |
| 5-Day PILOT Score ^Ω^ | 363 (86) | 5 (2, 8) | 68 (15) | 6 (3, 9) | 295 (71) | 5 (2, 8) | 0.2051 |
| 5-Day PILOT Score Maximum | 86 | 10 (6, 14) | 15 | 13 (9, 16) | 71 | 10 (6, 14) | 0.4174 |
| 5-Day PILOT Score Minimum | 86 | 2 (2, 4) | 15 | 2 (2, 5) | 71 | 2 (2, 4) | 0.8686 |
| In-Hospital Mortality | 292 | 78 (27%) | 54 | 16 (30%) | 238 | 62 (26%) | 0.5915 |
| GOSE | 225 | 4 (1, 8) | 43 | 3 (1, 8) | 182 | 4 (1, 8) | 0.5201 |
| GOSE <4 | 225 | 105 (47%) | 43 | 23 (54%) | 182 | 82 (45%) | 0.3188 |
| GOSE <5 | 225 | 120 (53%) | 43 | 25 (58%) | 182 | 95 (52%) | 0.4824 |
| GOSE >6 | 225 | 89 (40%) | 43 | 16 (37%) | 182 | 73 (40%) | 0.7265 |
|  |  |  |  |  |  |  |  |

ICP: Intracranial pressure, recorded within 5 days of admission; ^≠^ N: ICP measurements (participants); PILOT: Pediatric intensity level of therapy scale, within 5 days of admission; ^Ω^ N: PILOT score measurements (participants); GOSE: Glasgow outcome scale-extended recorded on first follow-up visit; IQR: interquartile range; * Significant P-values <0.05
